# RETRACTED: Alhakamy et al. Attenuation of Benign Prostatic Hyperplasia by Optimized Tadalafil Loaded Pumpkin Seed Oil-Based Self Nanoemulsion: In Vitro and In Vivo Evaluation. *Pharmaceutics* 2019, *11*, 640

**DOI:** 10.3390/pharmaceutics15122654

**Published:** 2023-11-22

**Authors:** Nabil A. Alhakamy, Usama A. Fahmy, Osama A. A. Ahmed

**Affiliations:** 1 Department of Pharmaceutics, Faculty of Pharmacy, King Abdulaziz University, Jeddah 21589, Saudi Arabia; uahmedkauedu.sa@kau.edu.sa (U.A.F.); osama71200@gmail.com (O.A.A.A.); 2 Department of Pharmaceutics & Industrial Pharmacy, Faculty of Pharmacy, Minia University, Minia 61519, Egypt

The journal retracts the article, “Attenuation of Benign Prostatic Hyperplasia by Optimized Tadalafil Loaded Pumpkin Seed Oil-Based Self Nanoemulsion: In Vitro and In Vivo Evaluation” [1].

Following publication, concerns were brought to the attention of the publisher regarding overlapping figures across two other publications [2,3], representing different experimental conditions. More specifically, Figure 2B in paper [1] is duplicated as Figure 2 in the published paper [2], and partially duplicated as Figure 1B in the published paper [3]. The authors commented that the TEM images in this article [2] are correct and that they were inadvertently duplicated in [1,3] due to a coding error of the data files. While the authors fully cooperated with the Editorial Office during the investigation, they were unable to satisfactorily explain the overlapping of figures and meet the required quality standards of raw images in order to consider a correction as per the journals original image requirements policy (https://www.mdpi.com/journal/pharmaceutics/instructions#oriimages). As a result, the Editorial Board and Editor-in-Chief were unable to confirm the reliability of the findings and subsequently decided to retract the paper. 

This retraction was approved by the Editor-in-Chief of *Pharmaceutics*.

The authors disagree with this retraction.
